# Hypoxia-induced nitric oxide production and tumour perfusion is inhibited by pegylated arginine deiminase (ADI-PEG20)

**DOI:** 10.1038/srep22950

**Published:** 2016-03-14

**Authors:** Natalie Burrows, Gaelle Cane, Mathew Robson, Edoardo Gaude, William J. Howat, Peter W. Szlosarek, R. Barbara Pedley, Christian Frezza, Margaret Ashcroft, Patrick H. Maxwell

**Affiliations:** 1 School of Clinical Medicine, Cambridge Institute for Medical Research, University of Cambridge, Cambridge Biomedical Campus, Hills Road, Cambridge, CB2 0XY, United Kingdom; 2 Metabolism and Experimental Therapeutics, Division of Medicine, University College London, 5 University Street, London, WC1E 6JF, United Kingdom; 3 Tumour Biology Group, UCL Cancer Institute, University College London, London WC1E 6BT, United Kingdom; 4 MRC Cancer Unit, University of Cambridge, Hutchison/MRC Research Centre, Box 197, Cambridge Biomedical Campus, Cambridge, United Kingdom, CB2 0XZ; 5 Histopathology/ISH, Cancer Research UK Cambridge Institute, University of Cambridge, Li Ka Shing Centre, Robinson Way, Cambridge, CB2 0RE, United Kingdom; 6 Centre for Molecular Oncology, Barts Cancer Institute, Queen Mary University of London, London, EC1M 6BQ, United Kingdom

## Abstract

The hypoxic tumour microenvironment represents an aggressive, therapy-resistant compartment. As arginine is required for specific hypoxia-induced processes, we hypothesised that arginine-deprivation therapy may be useful in targeting hypoxic cancer cells. We explored the effects of the arginine-degrading agent ADI-PEG20 on hypoxia-inducible factor (HIF) activation, the hypoxia-induced nitric oxide (NO) pathway and proliferation using HCT116 and UMUC3 cells and xenografts. The latter lack argininosuccinate synthetase (ASS1) making them auxotrophic for arginine. In HCT116 cells, ADI-PEG20 inhibited hypoxic-activation of HIF-1α and HIF-2α, leading to decreased inducible-nitric oxide synthase (iNOS), NO-production, and VEGF. Interestingly, combining hypoxia and ADI-PEG20 synergistically inhibited ASS1. ADI-PEG20 inhibited mTORC1 and activated the unfolded protein response providing a mechanism for inhibition of HIF and ASS1. ADI-PEG20 inhibited tumour growth, impaired hypoxia-associated NO-production, and decreased vascular perfusion. Expression of HIF-1α/HIF-2α/iNOS and VEGF were reduced, despite an increased hypoxic tumour fraction. Similar effects were observed in UMUC3 xenografts. In summary, ADI-PEG20 inhibits HIF-activated processes in two tumour models with widely different arginine biology. Thus, ADI-PEG20 may be useful in the clinic to target therapy-resistant hypoxic cells in ASS1-proficient tumours and ASS1-deficient tumours.

Recombinant pegylated-arginine deiminase (ADI-PEG20) degrades arginine and is currently in clinical trials for treating cancers auxotrophic for arginine due to loss of argininosuccinate synthetase (ASS1)^1^. Arginine is required for a number of biosynthetic pathways that contribute to cancer progression, including nitric oxide (NO) synthesis. Thus arginine-deprivation therapy inhibits NO-production, vascular endothelial growth factor (VEGF) expression, tumour perfusion, angiogenesis and growth^2^,^3^,^4^.

Hypoxia is a common feature of most solid tumours and represents an aggressive, therapy-resistant cellular compartment. Well-recognised effects of hypoxia include altered cell metabolism^5^,^6^, increased NO synthesis via induction of iNOS, and increased VEGF expression. These are orchestrated by the transcription factors HIF-1 and HIF-2, which regulate the expression of many genes involved in tumour biology^7^,^8^. Little is known about the effects of arginine-deprivation on hypoxic cancer cells and remarkably, the effects *in vivo* are unknown.

We reasoned that tumour regions poorly supplied with oxygen and nutrients, may make effective arginine-deprivation easier to achieve. We used the ASS1-positive HCT116 colon-carcinoma cell line, well established for forming hypoxic tumours, as a model to assess the effects of combining arginine-deprivation with hypoxia. We additionally assessed the effect of ADI-PEG20 on ASS1-deficient UMUC3 xenografts to compare the effects in ASS1-proficient and deficient tumours.

## Results

### ADI-PEG20 inhibits hypoxia-induced expression of HIF-α and iNOS

**Figure 1:**
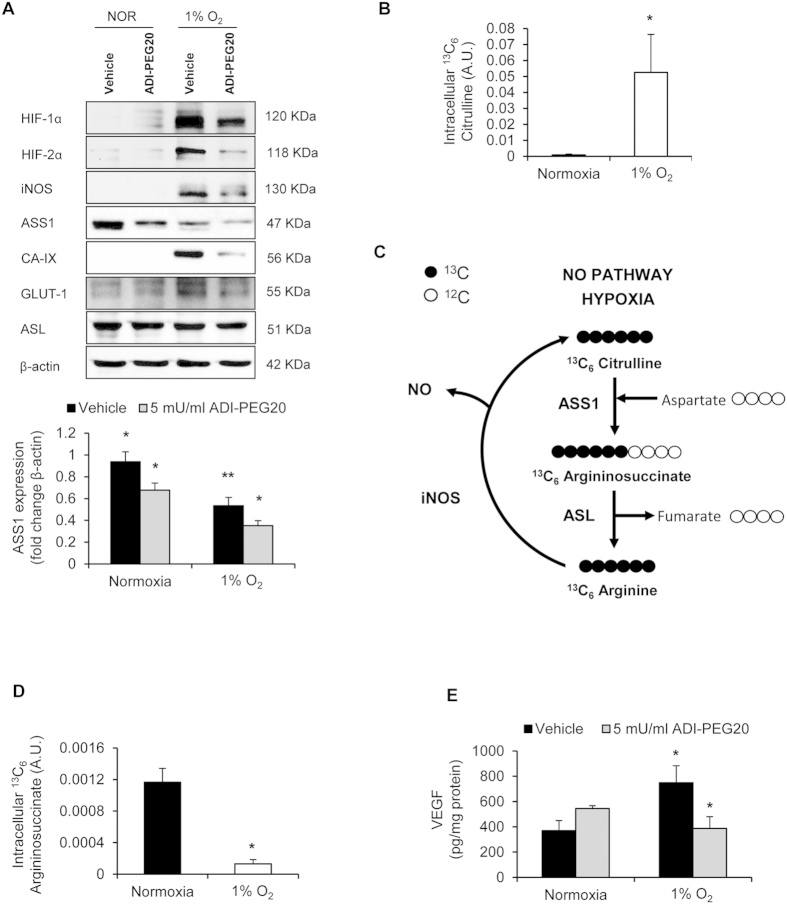
Under hypoxia, ADI-PEG20 inhibits HIF-α and iNOS and expression of VEGF. Both hypoxia and ADI-PEG20 inhibit ASS1 expression. (**A**) Western-blot analysis of HCT116 cells cultured for 24 h (normoxia/hypoxia) with vehicle (PBS) or 5 mU/ml ADI-PEG20. Under hypoxia, ADI-PEG20 inhibits expression of HIF-1α/HIF-2α/iNOS/CA-IX and GLUT-1. ADI-PEG20 reduces ASS1 expression under normoxia/hypoxia (*p < 0.05). Hypoxia alone reduces ASS1 expression 0.57fold (**p < 0.01). Densitometry of ASS1 expression relative to β-actin for 5 experiments is shown. ASL is unaffected. Blot is representative of 5 experiments. (**B**) Under hypoxia, ^13^C_6_ citrulline was increased (*p < 0.05) versus normoxia. Cells were incubated with ^13^C_6_ arginine for 24 h and the isotopologue distribution of ^13^C_6_ citrulline detected by LC-MS. (**C**) Schematic representation of the fate of ^13^C_6_ arginine in the NO pathway. Under hypoxia, iNOS converts ^13^C_6_ arginine into ^13^C_6_ citrulline and NO. ^13^C_6_ citrulline is then converted into ^13^C_6_ argininosuccinate and ^13^C_6_ arginine by ASS1 and ASL, respectively. (**D**) ^13^C_6_ argininosuccinate is reduced under hypoxia (*p < 0.05), indicative of reduced ASS1 activity. Cells were incubated with ^13^C_6_ arginine for 24 h and the isotopologue distribution of ^13^C_6_ argininosuccinate assessed. Note that the increase in ^13^C_6_ citrulline in hypoxia will be due to a combination of reduced ASS1 activity and iNOS induction. (**E**) Intracellular VEGF increases under hypoxia versus normoxia (*p < 0.05). 5 mU/ml ADI-PEG20 reduces VEGF under hypoxia (*p < 0.05). HCT116 cells were cultured under normoxia/hypoxia for 24 h and intracellular VEGF concentration determined by ELISA. Data represents the mean ± S.E.M. of 5 experiments.

To examine potential interactions between arginine-deprivation and hypoxia, we explored the effect of ADI-PEG20 on hypoxia-induced HIF-α protein. Under hypoxia, ADI-PEG20 reduced expression of HIF-1α, HIF-2α and downstream-target iNOS, which requires arginine as the substrate for NO synthesis (Fig. 1A). The inhibitory effects of ADI-PEG20 on HIF-activation were further confirmed by analysis of additional HIF-downstream targets, carbonic anhydrase-IX (CA-IX) and glucose transporter-1 (GLUT-1), both of which were reduced (Fig. 1A). Additionally, when arginine-deprivation was achieved using arginine-free media, hypoxia-induced HIF-activation was also inhibited (Supplementary Figure 1A). In support of these data, when HIF-α protein was stabilised under normoxia with dimethyloxaloylglycine (DMOG) that inhibits the oxygen-regulated prolyl hydroxylation of HIF-α^9^; arginine-deprivation therapy similarly reduced HIF-α protein (Supplementary Figure 1B).

Next, we examined the effect of hypoxia on arginine utilisation for NO production using stable-isotopologue tracing by LC-MS. HCT116 cells were cultured for 24 h under normoxia or hypoxia (1% O_2_) in the presence of uniformly labelled ^13^C-arginine, (^13^C_6_ arginine) and the isotopologue distribution of intracellular citrulline and argininosuccinate assessed. Under hypoxia, increased levels of ^13^C_6_ citrulline were observed, indicative of an active NO pathway (Fig. 1B,C). We additionally assessed the effect of hypoxia on key proteins regulating arginine metabolism. Hypoxia significantly inhibited ASS1-expression 0.57 fold (Fig. 1A) and activity; assessed by metabolomic analysis of ^13^C_6_ argininosuccinate (Fig. 1D). Since increased ^13^C_6_ citrulline (Fig. 1B) may also be due to reduced ASS1 activity under hypoxia; we further confirmed the existence of an active NO pathway via a NO ELISA, which showed increased NO in hypoxic-cell supernatants (Supplementary Figure 1C). Thus, increased ^13^C_6_ citrulline under hypoxia may be a combined effect of iNOS induction and reduced ASS1 activity.

### ADI-PEG20 inhibits hypoxia-induced activation of the NO pathway *in vitro*

Next, we assessed the effects of ADI-PEG20 on hypoxia-induced arginine utilisation for NO production. ADI-PEG20 converts arginine into equimolar amounts of citrulline and ammonia: Similar to previous observations^10^, ADI-PEG20 converted 99.7% of extracellular ^13^C_6_ arginine to ^13^C_6_ citrulline (Supplementary Figure 2A,B). Intracellular ^13^C_6_ arginine was significantly reduced by 94.4 ± 5.6% (normoxia) and 96.2 ± 5.2% (hypoxia: Supplementary Figure 2A) confirming that ADI-PEG20-treatment depleted the intracellular arginine pool, required for NO-production. Interestingly, ADI-PEG20 significantly inhibited ASS1 expression under normoxia and hypoxia, with the inhibitory effect under hypoxia being more severe (Fig. 1A). We did not observe any modulation of ASL by hypoxia or ADI-PEG20 (Fig. 1A).

As NO regulates VEGF and vice versa^11^,^12^,^13^ and given that VEGF is a HIF-downstream target^14^, we explored the effects of ADI-PEG20 on VEGF expression. Whilst ADI-PEG20 had little effect on normoxic VEGF, hypoxic-induced VEGF was significantly inhibited (Fig. 1E).

### ADI-PEG20 reduces HIF-α and ASS1 protein via inhibition of mTORC1 and activation of the unfolded protein response (UPR)

**Figure 2:**
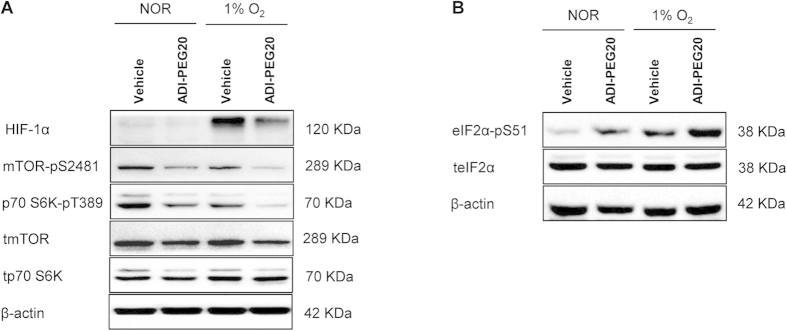
Under hypoxia, ADI-PEG20 markedly inhibits mTORC1 activity and activates the UPR (ascertained by eIF-2α-pS51 expression). (**A**) Hypoxia inhibits mTORC1 activity ascertained by reduced protein expression of mTOR-pS2481 and downstream target p70 S6K-pT389. 5mU/ml ADI-PEG20 also inhibits mTOR-pS2481 and p70 S6K-pT389 protein expression under normoxia and hypoxia; with the effect being more marked under hypoxia. Total (t) mTOR and tp70 S6K were unaffected. (**B**) Hypoxia and 5 mU/ml ADI-PEG20 independently activate the UPR (shown by increased eIF-2α-pS51 protein). In ADI-PEG20-treated cells, increased eIF-2α-pS51 is markedly higher in hypoxia versus normoxia, teIF-2α was unaffected. For (**A**,**B**), cells were cultured as described (Fig. 1A). β-actin was used as the loading control. Blots are representative of 3 experiments.

To explore the mechanism by which ADI-PEG20 influenced HIF-α and ASS1 protein expression; we considered the mammalian target of rapamycin complex 1 (mTORC1) and the unfolded protein response (UPR) pathways, which contribute to adaptation to hypoxia and nutrient-deprivation^15^. Hypoxia alone inhibited phosphorylation and activation of mTORC1 and its downstream target p70 S6 Kinase (p70 S6K), and activated the UPR ascertained by increased expression of phospho-eukaryotic translation-initiation-factor 2α (eIF2α-pS51): Under normoxia, ADI-PEG20 inhibited mTORC1 and p70 S6K activity and activated the UPR. However, under hypoxia these effects were much more marked (Fig. 2A,B). We did not see an effect of ADI-PEG20 on MAPK signalling (data not shown), another pathway regulating HIF-α protein expression. Thus, the marked inhibitory effect of ADI-PEG20 on ASS1 and HIF-α protein under hypoxia may be due to inhibition of mTORC1 and activation of the UPR.

**Figure 3:**
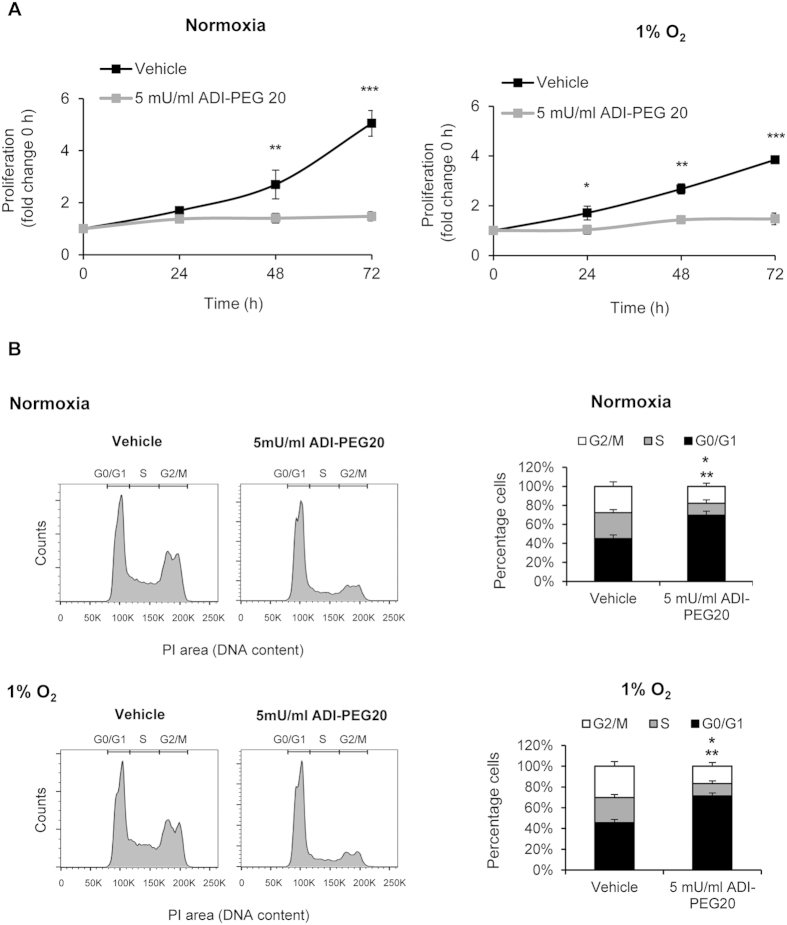
ADI-PEG20 inhibits proliferation equally under normoxia/hypoxia and induces a G1 arrest. (**A**) ADI-PEG20 inhibits proliferation (assessed by MTT assay) under normoxia/hypoxia (*p < 0.05, **p < 0.01, ***p < 0.001 versus vehicle). (**B**) ADI-PEG20 increases percentage cells in G1/G0 (**p < 0.01) and reduces percentage cells in S, G2/M phase (*p < 0.05) under normoxia/hypoxia. Histograms showing cell cycle distribution and quantification of percentage cells in G1/G0, S and G2/M are shown. Cells were cultured for 24 h with vehicle or 5 mU/ml ADI-PEG20 under normoxia/hypoxia (1% O_2_). Data represents the mean ± S.E.M. of 3–4 experiments.

We next assessed the effect of ADI-PEG20 on proliferation. ADI-PEG20 inhibited proliferation similarly in both normoxia and hypoxia (Fig. 3A) and induced a G1 arrest after 24 h (Fig. 3B), which was also seen at 48 and 72 h (not depicted). These data are in line with the inhibitory effect of ADI-PEG20 on the mTORC1 downstream-target p70 S6K, which regulates G1-S phase transition^16^. We further observed that the antiproliferative effects of ADI-PEG20 on parental and HCT116 cells defective for p53 function (p53−/−) was similar in both normoxia and hypoxia (Supplementary Figure 3A), implying that the antiproliferative effect of ADI-PEG20 may not require p53 in this model system.

### Confirmation of ASS1 functional activity in HCT116 cells

We have shown in HCT116 cells that ASS1 is expressed and active (by the formation of ^13^C_6_ argininosuccinate; Fig. 1A,D). However, we wanted to confirm that the ASS1 activity shown in our metabolomics studies translated to a significant functional effect: To ascertain this, we assessed the ability of ASS1 to rescue cell proliferation in the presence of its substrate citrulline, and ammonium chloride in arginine-free media. We compared this with known ASS1-proficient (HeLa) and ASS1-deficient (UMUC3) cell lines. Both HeLa and HCT116 cell proliferation was significantly increased in arginine-free media supplemented with citrulline, whilst no rescue was observed in ASS1-deficient UMUC3 cells (Supplementary Figure 3B). These data confirm that ASS1 is functionally active in HCT116 cells.

### ADI-PEG20 inhibits growth in both ASS1-proficient and deficient tumours

**Figure 4:**
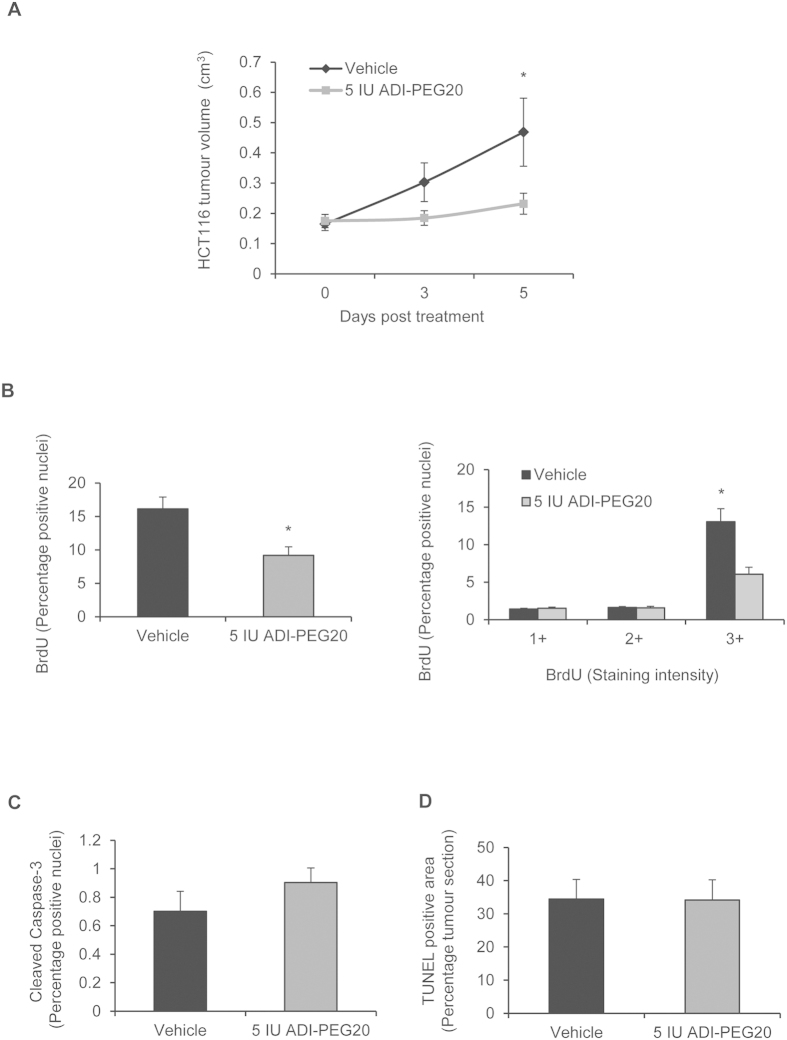
ADI-PEG20 inhibits HCT116 tumour growth. (**A**) 5IU ADI-PEG20 inhibits tumour growth on days 3 and 5 post therapy by 39.1% ± 7.4% S.E.M and 50.5% ± 7.4% S.E.M, respectively (*p < 0.05 versus vehicle (PBS) treated mice). (**B**) Percentage BrdU positive-nuclei and nuclear expression are lower in ADI-PEG20-treated tumours (*p < 0.05). Nuclear expression was scored as weak (+1), medium (+2) and intense (+3). See methods. (**C**) Percentage nuclei positive for cleaved caspase-3 and (**D**) TUNEL-positive cells did not significantly differ. Data represents the mean ± S.E.M. of 5 mice per group.

To determine if the inhibitory effects of ADI-PEG20 on the HIF-pathway translated *in vivo*, mice bearing size-matched HCT116 xenografts were treated with 5IU ADI-PEG20 (i.p.) or an equal volume of vehicle. Tumours were excised 5 days later for analysis. No loss of weight or impaired health (data not shown) was observed. ADI-PEG20-mediated depletion of arginine was confirmed by metabolomic analysis of plasma arginine and citrulline (Supplementary Figure 4). ADI-PEG20 reduced tumour growth on days 3 and 5 following the start of treatment by 39.1% ± 7.4% (S.E.M) and 50.5% ± 7.4% (S.E.M), respectively (Fig. 4A). Inhibition of tumour growth was paralleled by a significant reduction in proliferating cells, assessed via immunohistochemical detection of BrdU incorporation (Fig. 4B, Supplementary Figure 5A). Apoptotic cells were assessed by analysis of cleaved-caspase-3 expression and TUNEL-positive cells (Fig. 4C,D, Supplementary Figure 5B); there was not a statistically significant difference in cleaved-caspase-3, TUNEL-positive cells or necrotic fraction (Supplementary Figure 6).

### ADI-PEG20 inhibits hypoxia-induced NO-production *in vivo*

**Figure 5:**
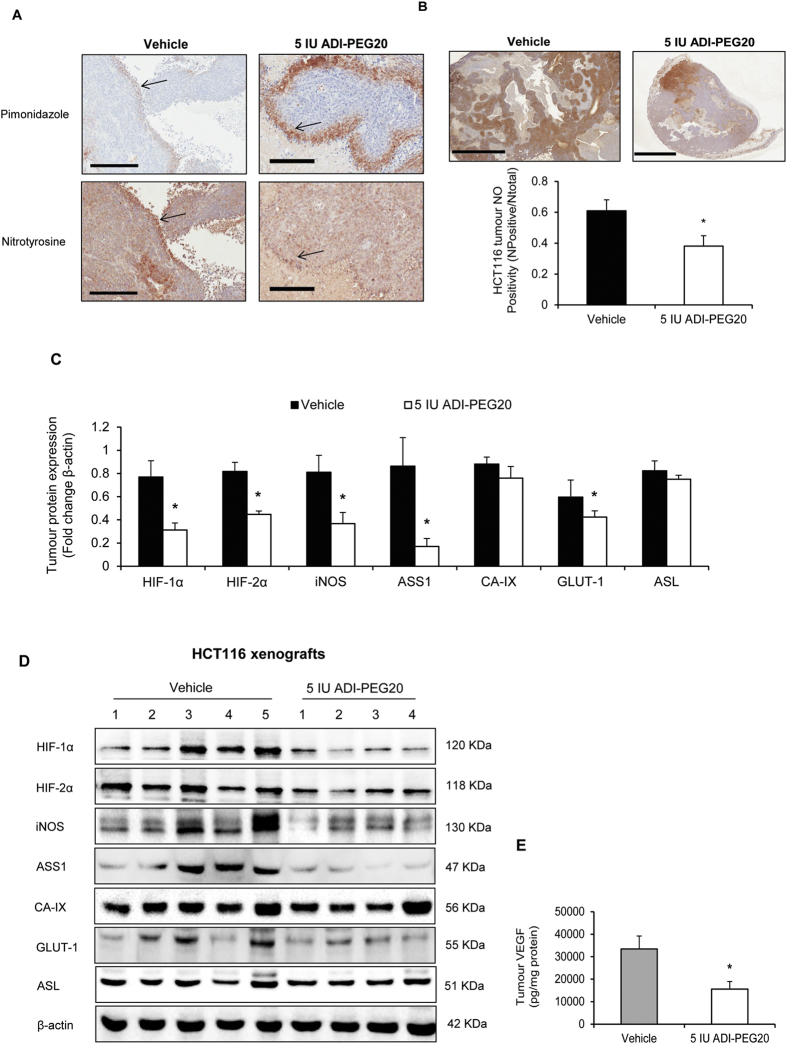
ADI-PEG20 reduces hypoxia-induced and total NO and inhibits expression of HIF-α, iNOS, ASS1 and VEGF in HCT116 xenografts. (**A**) Serial sections were incubated with primary antibodies against nitrotyrosine and pimonidazole to detect NO in hypoxic regions (brown staining). NO levels are elevated in hypoxic regions and reduced in ADI-PEG20 treated tumours (indicated by arrows). (**B**) ADI-PEG20-treated tumours exhibit lower total nitrotyrosylated protein expression than controls (*p < 0.05). Total nitrotyrosine expression varied within both vehicle and ADI-PEG20 treated tumours; expression ranged from weak to intense and localization from diffuse to focal. NO expression was analysed as described (methods). Positivity represents number of positive pixels/total pixels. Scale-bar for (**A**) 400 μm, for (**B**) 2 mm. (**C**) Densitometry analysis of western-blot shown in (**D**) consisting of 5 vehicle-treated and 4 ADI-PEG20-treated HCT116-tumours. ADI-PEG20 reduces HIF-1α/HIF-2α/ iNOS/ASS1 and GLUT-1 expression (*p < 0.05). CA-IX and ASL are unaffected. (**E**) VEGF is reduced in ADI-PEG20-treated tumours versus controls (*p < 0.05), assessed by ELISA. Data represents the mean ± S.E.M. of 5 mice per group.

We next assessed the effects of ADI-PEG20 on NO-production by analysing expression of nitrotyrosylated proteins via IHC. NO and metabolites readily react with tyrosine residues to form nitrotyrosine, providing an indicator of NO-production. HCT116 cell pellets pre-treated with 3 mM peroxynitrite were used as a positive control for nitrotyrosine staining (Supplementary Figure 7). To determine if regions of hypoxia were associated with increased NO-production *in vivo*, animals were given pimonidazole which forms adducts selectively in hypoxia. Serial tumour sections were incubated with antibodies against pimonidazole and nitrotyrosine: in regions that stained positive for pimonidazole, nitrotyrosine signal was elevated, indicative of hypoxia-induced NO-production. In ADI-PEG20-treated tumours, NO signal was lower in hypoxic tumour regions when compared with controls (Fig. 5A). The amount of nitrotyrosine signal was variable in vehicle and ADI-PEG20-treated tumours, but ADI-PEG20 clearly reduced overall nitrotyrosine signal (Fig. 5B), confirming inhibition of NO-production in ASS1-proficient tumours *in vivo*.

### ADI-PEG20 impairs vascular perfusion and increases hypoxic fraction, whilst maintaining an inhibitory effect on HIF-activation, iNOS and ASS1 expression *in vivo*

We showed *in vitro* that ADI-PEG20 inhibited hypoxia-induced NO by additional mechanisms besides the direct effect of arginine-depletion; that is by reducing expression of HIF-α, iNOS and inhibiting ASS1 expression/activity. We next examined which of these may contribute to decreasing NO *in vivo*. As observed *in vitro*, ADI-PEG20 significantly reduced expression of HIF-1α, HIF-2α, iNOS, VEGF and GLUT-1, although interestingly we observed little effect on CA-IX. ASS1-expression was also reduced in ADI-PEG20-treated tumours with little effect seen on ASL (Fig. 5C–E).

**Figure 6:**
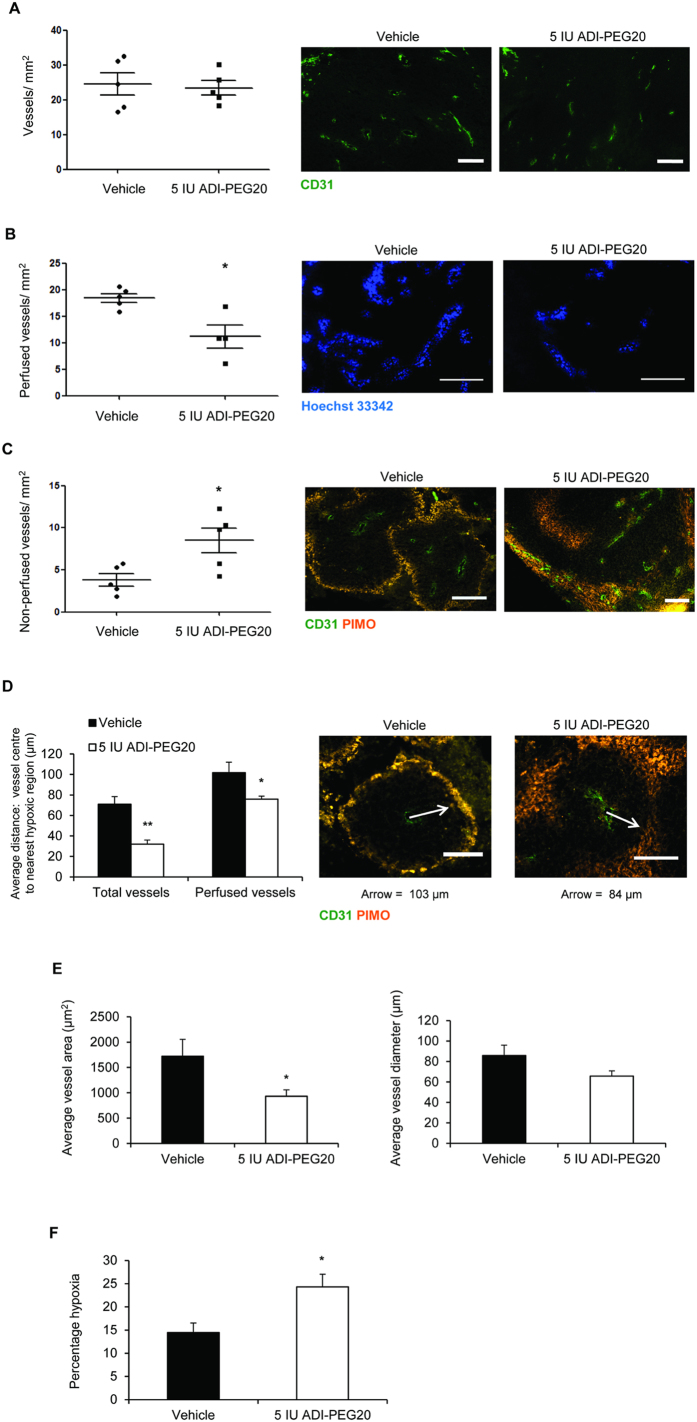
ADI-PEG20 reduces vascular perfusion and vessel size in HCT116 xenografts. (**A**) Total vessels are similar between groups. In ADI-PEG20-treated tumours (**B**) perfused vessels are reduced (*p < 0.05) and (**C**) non-perfused vessels are increased (*p < 0.05). (**D**) Average distance from vessel centre to nearest hypoxic region is reduced (*p < 0.05, **p < 0.01) and (**E**) average vessel area is reduced (*p < 0.05). Average vessel diameter was reduced (not significantly). (**F**) Percentage hypoxia is increased in ADI-PEG20-treated tumours (*p < 0.05). Total pimonidazole-positive area was quantified as percentage of overall tumour area. For (**A**–**F**), frozen sections were incubated with antibodies against CD31 (green) to detect vessels and PIMO (orange) to detected hypoxic regions and non-perfused vessels (see methods). Hoechst 33342-positive perfused vessels (blue) were counted in three fields of view at x200 magnification prior to fixation and immunofluorescence analysis. Total vessels were calculated/mm^2^ tumour section. Non-perfused vessels were quantified as those with no pimonidazole free areas between the vessel and surrounding tissue. Degree of perfusion was quantified by measuring average distance from the vessel centre to the nearest hypoxic region. Analysis was performed on at least 2 sections per tumour. Data represents the mean ± S.E.M. of 4–5 mice per group. Scale-bar for (**A-C**), 200 μm, scale-bar for (**D**), 100 μm.

We next assessed if inhibition of tumour growth may in part be due to alterations in the vasculature as a result of reduced HIF/NO-signalling by ADI-PEG20. Immunofluorescence was performed with antibodies against CD31 to identify vessels, Hoechst 33342 to identify perfused vessels and pimonidazole for assessment of hypoxia. Whilst total vessel count per unit area did not differ between groups (Fig. 6A), tumours treated with ADI-PEG20 had significantly fewer perfused vessels (Fig. 6B) and significantly more non-perfused vessels (Fig. 6C). In accord with this, the average distance from vessel centre to the closest hypoxic region was significantly reduced (Fig. 6D). Average vessel size was also reduced (Fig. 6E). Consistent with reduced perfusion, total hypoxic fraction was significantly higher (Fig. 6F).

**Figure 7:**
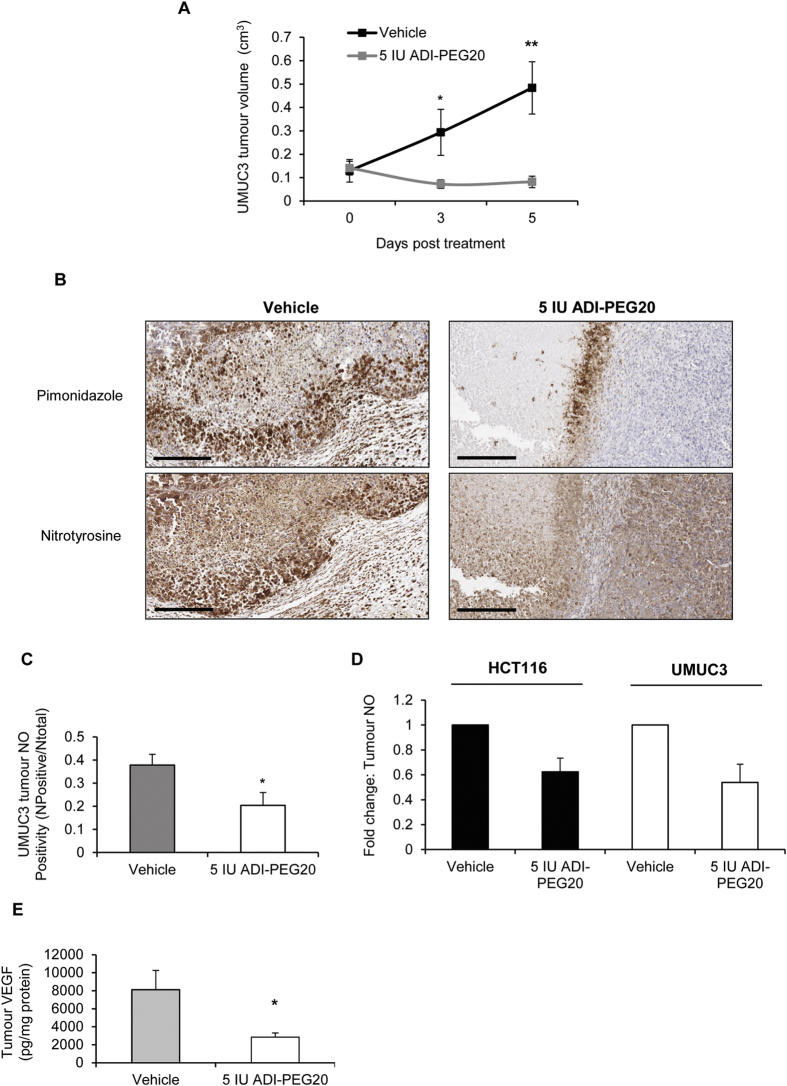
ASS1-deficient UMUC3 xenografts are more sensitive to the antiproliferative effects of ADI-PEG20. However, ADI-PEG20 inhibits hypoxia-induced/total NO and tumour VEGF similarly to that observed in HCT116 xenografts. (**A**) ADI-PEG20 inhibits tumour growth on days 3 and 5 post therapy by 75.4% ± 6.1% S.E.M. and 83.1% ± 5% S.E.M., respectively (*p < 0.05, **p < 0.01 versus vehicle-treated mice). (**B**) Serial sections were incubated with primary antibodies against nitrotyrosine and pimonidazole to detect NO production in hypoxic regions (brown staining). NO levels are elevated in hypoxic regions and reduced in ADI-PEG20-treated tumours. (**C**) ADI-PEG20-treated tumours have lower total nitrotyrosylated protein expression than controls (*p < 0.05). Positivity represents number of positive pixels/total pixels. (**D**) Fold reduction in tumour NO in ADI-PEG20-treated mice is similar between tumour types. NO levels were reduced by 0.38 fold compared to controls, in HCT116 xenografts, and 0.46 fold in UMUC3 xenografts. (**E**) Tumour VEGF is reduced in ADI-PEG20 treated mice (*p < 0.05). Data represents the mean ± S.E.M. of 5 mice per group. Scale-bar for (**B**), 400 μm.

ADI-PEG20 is in clinical trials for use in patients with ASS1-deficient tumours. We therefore determined if ADI-PEG20 had similar effects on hypoxia-induced NO-synthesis and vascular function in ASS1-deficient UMUC3 xenografts. As anticipated, UMUC3 tumours were more sensitive to the antiproliferative effects of ADI-PEG20, with growth inhibition reaching 75.4% ± 6.1% (day 3) and 83.1% ± 5% (day 5) of controls (Fig. 7A). Similar to our observations in HCT116-xenografts, nitrotyrosine signal was elevated in pimonidazole positive regions indicating hypoxia-induced NO-production in UMUC3 tumours, which was reduced by ADI-PEG20 (Fig. 7B). Total nitrotyrosine signal was also reduced (Fig. 7C). Interestingly, the reduction in total nitrotyrosine expression was similar between ADI-PEG20-treated HCT116 and UMUC3 tumours (Fig. 7D). VEGF levels were approximately four times lower in UMUC3 versus HCT116 tumours. Nevertheless, a similar inhibitory effect by ADI-PEG20 was observed in UMUC3 tumours (Fig. 7E).

**Figure 8:**
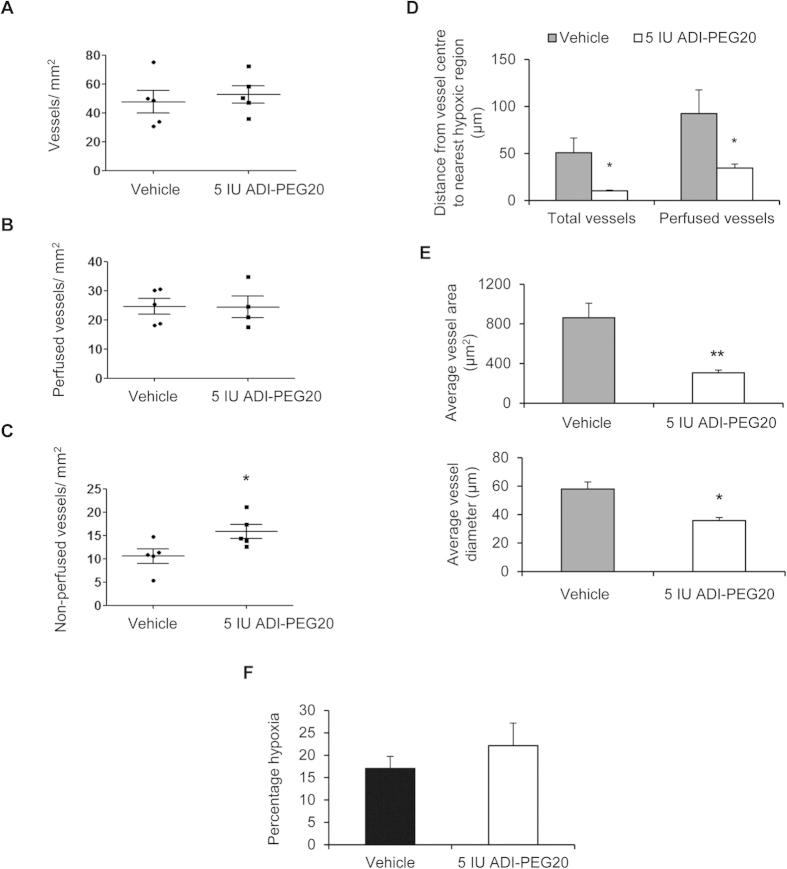
ADI-PEG20 reduces vascular perfusion and vessel size in UMUC3 xenografts. (**A**) Total number of vessels and (**B**) perfused vessels are similar between groups. In ADI-PEG20-treated tumours, (**C**) non-perfused vessels are increased (*p < 0.05) and (**D**) average distance from vessel centre to nearest hypoxic region is reduced (*p < 0.05). (**E**) Average vessel area and diameter are also reduced (*p < 0.05, **p < 0.01). (**F**) Percentage hypoxia is increased (not significantly). Analysis was performed as described (Fig. 6). Data represents the mean ± S.E.M. 4–5 mice per group.

In ADI-PEG20-treated UMUC3 tumours, vessel number per unit area did not differ (Fig. 8A,B). However, the number of non-perfused vessels was increased, and perfusion and vessel size were reduced (Fig. 8C–E) indicating a similar inhibitory effect on vascular perfusion to that seen in HCT116 tumours. In these tumours, the increase in hypoxic fraction did not reach statistical significance (Fig. 8F).

## Discussion

Arginine is required for specific hypoxia-mediated processes that contribute to tumour progression. This combined with the fact that hypoxic regions have a limited blood and nutrient supply, led us to hypothesise that arginine-deprivation therapy may be useful in targeting hypoxic-cancer cells, even in ASS1-expressing tumours. We used ADI-PEG20 because of its long half-life (7 days), low immunogenicity and good tolerability in clinical trials^1^. We show that ADI-PEG20 inhibits HIF-signalling and hypoxia-induced NO *in vitro* and in two tumour models with differing ASS1-expression.

There is limited data concerning the effect of arginine-deprivation on hypoxic cancer cells. One study showed that under normoxia, arginine-deprivation inhibited basal HIF-1α expression in ASS1-deficient melanoma cells^17^. We considered that a key unanswered question was whether arginine-deprivation altered activation of HIF in hypoxia. We found that under hypoxia, HIF-1α protein expression was indeed inhibited both by ADI-PEG20 and in arginine-free media, and we further report an inhibitory effect on HIF-2α protein expression. This was paralleled by a decrease in HIF signalling ascertained by reduced expression of the HIF downstream-targets CA-IX and GLUT-1. HIF-1 has been reported to inhibit ASS1 expression in ASS1-deficient melanoma cells^17^, which encouraged us to evaluate the effect of hypoxia on ASS1 expression in ASS1-expressing (HCT116) cancer cells. Indeed, we found that hypoxia reduced both ASS1 protein and activity. This together with reduced HIF-activation would be predicted to render hypoxic cells more sensitive to arginine-deprivation.

In general, ASS1-proficient tumours have been considered to be unsuitable for arginine-deprivation therapy. Here we show that HCT116 cells that express functionally active ASS1 are sensitive to ADI-PEG20. Interestingly, ADI-PEG20 did not increase ASS1 protein in the time frames reported in melanoma cells. In fact, we observed the opposite: ADI-PEG20 reduced ASS1 protein, which importantly, was more marked under hypoxia. This may further contribute to increased sensitivity to ADI-PEG20 in hypoxic tumour regions. That ADI-PEG20 can be effective in cells expressing ASS1 is supported by studies showing that arginine-deprivation inhibited ASS1 activity in some ASS1-expressing cell lines^18^ and inhibited cell proliferation in ASS1-positive retinoblastoma cells^19^. In this study, ASS1 activity was relatively low, despite high levels of protein. How sensitive individual cancers are to ADI-PEG20 will likely be influenced by the actual level of ASS1 activity, but our results imply that ADI-PEG20 could be clinically effective in tumours deemed ASS1-positive, by targeting hypoxic regions.

To explore the mechanism of inhibition of HIF-α and ASS1 protein, we assessed the effect of ADI-PEG20 on two pathways that inhibit protein synthesis in response to hypoxia and nutrient-deprivation; mTORC1 and the UPR. Previously arginine-deprivation therapy has been shown to inhibit mTORC1 and activate eIF-2α under normoxia^20^,^21^. Similarly, ADI-PEG20 inhibited mTORC1 and activated the UPR in normoxia, but importantly, these effects were much more marked in hypoxia, consistent with combining environmental stressors known to modulate these pathways. The mTORC1 and UPR pathways are linked via a negative feedback loop involving UPR-mediated inactivation of insulin receptor substrate 1 (IRS1), a positive regulator of mTORC1 activity^22^. This may contribute to the marked inhibition of mTORC1 and downstream-target p70 S6K, observed in ADI-PEG20-treated hypoxic cells.

Arginine is an obligate substrate for NO-synthesis, and arginine-deprivation therapy has been shown to inhibit NO-production, tumour-perfusion, angiogenesis and VEGF production^2^,^4^. To our knowledge, no previous reports exist on the effects of arginine-deprivation on hypoxia-signalling, hypoxia-induced NO, VEGF and the functional consequences of this, in both cells and tumours. *In vitro* we observed a marked inhibition of hypoxia-induced NO-production, which involved other mechanisms besides the direct effect of substrate depletion: Hypoxia-induced iNOS expression was inhibited in ADI-PEG20-treated cells, likely as a result of reduced HIF-activation. Additionally, ADI-PEG20, along with hypoxia, synergistically reduced ASS1-expression. This essentially makes hypoxic cells auxotrophic for arginine and dependent on an extracellular supply in terms of NO synthesis. This dependency is further enhanced since iNOS produces high levels of NO over prolonged periods and thus requires arginine from extracellular sources^23^,^24^. Collectively, these properties are likely to contribute to the marked inhibitory effect of ADI-PEG20 on hypoxia-induced NO, both in cells and tumours.

NO modulates expression of VEGF and vice versa^11^,^12^,^13^. Additionally, VEGF is a downstream HIF-target and a key modifier of vascular function^14^. We observed that ADI-PEG20 inhibited hypoxia-induced VEGF. In contrast, basal VEGF was unaffected suggesting that inhibition of hypoxia-induced VEGF is likely a result of the inhibitory effect of ADI-PEG20 on HIF.

To determine if our findings translated *in vivo*, HCT116 xenografts were treated with 5 IU ADI-PEG20 for 5 days. We chose this model as it is well established for forming hypoxic tumours. Furthermore, the relative ease of access to subcutaneous tumours simplifies growth measurement and facilitates rapid excision of tumours, allowing accurate assessment of HIF-α proteins, which is challenging due to their short half-life (~5 min)^25^. We chose to excise tumours 5 days post treatment because this time frame is established as suitable for assessing effects of agents that alter vascular function^26^,^27^.

We show for the first time an inhibitory effect of ADI-PEG20 on hypoxia-signalling, ASS1 and hypoxia-induced NO in tumours; ADI-PEG20 inhibited proliferation and expression of HIF-α, iNOS, ASS1, VEGF and GLUT-1 protein. Interestingly, CA-IX was not detectably reduced *in vivo*, which may reflect the long half-life of this protein^28^.

Importantly, we observed that NO in hypoxic tumour regions, was inhibited by ADI-PEG20. NO is a major physiological regulator of vascular function, and reducing systemic NO levels in patients could have adverse effects on blood pressure and heart rate. Importantly, in preclinical and clinical studies in which ADI-PEG20 was shown to reduce plasma NO, detrimental effects on blood pressure and heart rate were not observed^29^,^30^,^31^. This may be because endothelial (e)NOS can obtain adequate arginine for NO-synthesis via ASS1, whereas iNOS, is dependent on extracellular supply^23^,^24^. Thus hypoxia-induced NO in tumours appears highly sensitive to inhibition by ADI-PEG20, and this is unlikely to have detrimental side effects related to systemic NO depletion.

To assess if ASS1-proficient and ASS1-deficient tumours responded differently to ADI-PEG20 in the context of hypoxia-induced NO, studies were performed in ASS1-deficient UMUC3 xenografts. As expected, UMUC3 tumours were more sensitive to the antiproliferative effects of ADI-PEG20 compared to HCT116 xenografts. However, the inhibitory effect of ADI-PEG20 on hypoxia-induced NO, total NO and VEGF were similar. These data imply that tumours with widely different arginine biology could be susceptible to the effects of arginine-deprivation on hypoxia-induced responses. Potentially this could be combined with anti-proliferative therapy targeted to the specific tumour, with ADI-PEG20 inhibiting hypoxia-signalling.

Consistent with the marked effect on tumour NO and VEGF; ADI-PEG20 strikingly reduced blood vessel patency. There is conflicting and controversial data on the net effects of agents that reduce vascular perfusion. Reduced blood flow would lead to nutrient-starvation and tumour regression. However, drugs that reduce vascular-perfusion will exacerbate hypoxia, increasing HIF-activation and promoting tumour progression, metastasis and therapy-resistance. Excitingly we show that despite an increase in hypoxic fraction in ADI-PEG20-treated HCT116 tumours, HIF-activation was inhibited. Furthermore, when drugs that alter vascular-function are combined with another treatment modality, the therapeutic response is usually enhanced. Indeed, this has been shown with arginine-deprivation therapy; when combined with radiotherapy, a synergistic effect has been observed in preclinical cancer models^26^,^32^.

ADI-PEG20 may be an efficacious therapy for targeting the hypoxic tumour compartment. This is important since hypoxic tumour cells are inherently resistant to therapy and contribute to tumour progression. As ADI-PEG20 inhibited targets downstream of HIF, it is likely to be effective in inhibiting additional hypoxia-mediated processes involved in tumour progression. HIF-activation can occur in the absence of hypoxia as a result of loss of von Hippel Lindau function^33^ or activated growth factor pathways^34^ broadening the contexts in which inhibiting HIF with ADI-PEG20 may be useful. Taken together, our data provide a rationale for extending clinical studies of ADI-PEG20 to selected human cancers that express ASS1 and may improve response when combined with another anti-cancer therapy.

## Materials and Methods

### Cell culture

Cell lines of human colon-carcinoma (HCT116) and human bladder transitional-cell carcinoma (UMUC3) were maintained in RPMI-1640 medium. For functional studies, cells were cultured in SILAC-media (RPMI-1640: Sigma-Aldrich Company Ltd, Dorset, UK) supplemented with 0.05 g/L leucine, 0.04 g/L lysine, 0.2 g/L arginine, 10% dialyzed FCS. Cells were treated with 5 mU/ml ADI-PEG20 (Polaris Pharmaceuticals, Inc, San Diego, USA) or vehicle (phosphate-buffered saline; PBS) in normoxia/hypoxia (1% O_2_) for 24 h, unless stated otherwise.

### Exposures to hypoxia

Cells were placed in a GalaxyR tissue-culture incubator (Wolf Laboratories Ltd, York, UK) and gassed with 5%CO_2_:1%O_2_:N_2_ balance, or a hypoxic chamber (Don Whitley Scientific Ltd, Shipley, UK and Ruskinn Technology Ltd, Bridgend, UK) and gassed with 5%CO_2_:1%O_2_:N_2_ balance.

### U-^13^C-Arginine labelling, metabolic extraction of cells and media and metabolite quantification

Protocols used are previously described^10^. Briefly, cells were incubated in SILAC-media containing 0.4 mM U-^13^C-Arginine for 24 h in normoxia/hypoxia and 50 μl media was sampled and diluted 1:160 in pre-chilled extraction buffer (ES; 30%acetonitrile, 50%methanol, 10 ng/ml2-[4-(2-hydroxyethyl)piperazin-1-yl] ethanesulfonic acid (HEPES)), for extracellular metabolite analysis. For intracellular analysis, cells were washed with PBS and lysed in 1 ml ES per 1 × 10^6^ cells. Lysates were vortexed for 15 min (4 °C), then centrifuged (13000 rpm) for 10 min (4 °C). Media/supernatants were analysed by liquid chromatography-mass spectrometry (LC-MS). Media incubated in the absence of cells was used as a reference. Quantification of metabolites was performed as described^10^.

### Western-blotting

Cells and tissues were sonicated in 50 mM TRIS-HCL pH7.4/120 mM NaCl/5 mM EDTA/0.5%NP40/1 mM DL-Dithiotheitol/1 mM Phenylmethylsulfonyl fluoride/2 mM sodium orthovanadate/2 mM NaF/20 mM β-glycerol phosphate/5 mM Sodium pyrophosphate/complete Mini EDTA-free Protease Inhibitor Cocktail Tablet, as described^35^. Blots were imaged on a Chemidoc MP Imaging system (Bio-rad Laboratories Ltd, Hemel Hempstead, UK). Protein expression was quantified relative to the loading control protein β-actin by densitometry analysis using ImageJ.

### VEGF ELISA

Intracellular VEGF concentrations were calculated using the DuoSet ELISA kit (R&D Systems).

### Cell cycle analysis

Harvested cells were washed in ice-cold PBS, fixed in 70% ethanol (2 h at 4 °C) and washed in PBS. Cells were stained with propidium iodide (PI) solution (25 μg/ml PI, 20 μg/ml RNase A/T1, PBS) for 30 min at 37 °C, processed on a Becton-Dickinson Fortessa Analyser and analysed using FlowJo software.

### Cytotoxicity assay

Proliferation was assessed by MTT assay and calculated as fold change of 0 h absorbance.

### Tumour xenografts

HCT116 and UMUC3 cells were implanted subcutaneously (0.1 ml of 5 × 10^7^/ml PBS) in SCID mice (aged 8–12 weeks). Treatment commenced once tumours reached ~0.2 cm^3^. Tumour volume was calculated as: (length × breadth × depth × (π/6) = volume (cm^3^). Mice received 5 IU ADI-PEG20 or vehicle (PBS) via intraperitoneal injection (i.p.). Tumours were excised 5 days later for analysis. 2 h prior to sacrifice, mice received Bromodeoxyuridine (BrdU; i.p., 1 mg/ml, dosed at 1 ml/100 g) and pimonidazole (60 mg/kg i.p.). 30 sec prior to sacrifice, the perfusion marker Hoechst 33342 was administered intravenously (i.v., 0.1 ml of a 6 mg/ml stock). All procedures were ethically approved by the University College London Animal Welfare and Ethical Review Body and complied with the Scientific Procedures Act 1986 and Guidelines for the Welfare and Use of Animals in Cancer 2010^36^, under the authority of Home Office licenses PPL70/7439 and PPL 70/7309.

### Immunofluorescence (IF)

Frozen tumour sections (8 μm) were imaged under a fluorescent microscope to quantify Hoechst-positive (perfused) vessels. Sections were fixed in acetone at −20 °C (10 min) and blocked with 10% horse serum, PBS (15 min). Sections were incubated with primary antibody in a humidified container for 18 h (4 °C), washed, incubated with a secondary Alexafluor-labelled antibody, washed, counterstained with 4′,6-diamidino-2-phenylindole (DAPI) and mounted in fluorescent mounting media (DAKO UK Ltd, Ely, UK).

### Immunohistochemistry (IHC)

3 μm formalin-fixed sections were stained on a BondMax Autostainer (Leica Microsystems (UK) Ltd, Milton Keynes, UK). For Cleaved Caspase-3, antigen retrieval was performed using Tris/EDTA solution pH9 (Bond ER2: 100 °C) and sections incubated with primary antibody for 15 min, followed by an 8 min incubation with biotinylated donkey anti-rabbit antibody (Stratech Scientific Limited, Newmarket, UK) and an 8 min incubation in Streptavidin-HRP (Leica Intense R kit) at room temperature). Antibody-antigen complexes were visualised with diaminobenzidine, using copper enhancement, and counterstained. For BrdU, the same protocol was used, with addition of 15 min incubation in 2 M HCL and antigen retrieval in Proteinase K at 25 ug/ml for 10 mins (37 °C). Slides were dehydrated and mounted (Leica ST5020 attached coverslipper CV5030). Pimonidazole and nitrotyrosine IHC was performed using the EnVision detection system (DAKO). Appropriate IgG antibodies were used as concentration-matched negative controls. The terminal deoxynucleotidyl transferase–mediated dUTP nick end labelling (TUNEL) *in situ* cell death detection kit, Fluorescein (Roche Diagnostics Ltd, Burgess Hill, UK) was additionally used for apoptotic cell detection.

### Visualisation and quantitation of tumour sections

Immuno-fluorescently labelled sections were digitized on a Leica Ariol SL-50 (Leica) at 20× magnification using Ariol software (Leica). For vessel analyses, three circular regions (diameter 100 μm) were randomly assigned onto the tumour sections and analysis was performed within these regions. For quantification of pimonidazole-positive regions, positive staining was determined by a threshold >71 grey levels.

IHC-labelled slides were digitized at 20× magnification on an Aperio XT (Leica) and analysed using Aperio/Spectrum v10.2.2.2317 software. BrdU expression was quantified using Aperio nuclear analysis v9, which split individual nuclei into negative, 1+, 2+, 3+ bins based on the average grey scale of individual nuclei, with the positive bins representing weak, intermediate and strong staining respectively. Grey scale values were 255-210 (negative), 210-188(1+), 188-162(2+) and 162-0(3+). Cleaved caspase-3 expression was quantified as for BrdU, with thresholds set so that strong (+3) staining was scored as positive. For nitrotyrosylated protein analysis; the entire tumour area was delineated and a negative mask applied to necrotic regions. Positive-pixel count analysis (Aperio v9) was applied using a Hue Value of 0.1, Hue Width of 0.5 and Colour Saturation Threshold of 0.15. Pixels were placed into three bins, as detailed above. The bins were 255-220 (negative), 220-175(1+), 175-100(2+) and 100–0(3+).

### Primary antibodies

See supplementary table 1.

### Statistical Analysis

Student’s *t* test and one-way ANOVA with Tukey *post hoc* test were used.

## Additional Information

**How to cite this article**: Burrows, N. *et al.* Hypoxia-induced nitric oxide production and tumour perfusion is inhibited by pegylated arginine deiminase (ADI-PEG20). *Sci. Rep.* **6**, 22950; doi: 10.1038/srep22950 (2016).

## Supplementary Material

Supplementary Information
