# Supplementary material for: Hypoxia-induced nitric oxide production and tumour perfusion is inhibited by pegylated arginine deiminase (ADI-PEG20)
Source: Sci Rep. 2016 Mar 14;6:22950. doi: 10.1038/srep22950 (PMC4789736; doi:10.1038/srep22950)
Supplement: Supplementary Information [file srep22950-s1.pdf]

Hypoxia-induced nitric oxide production and tumour perfusion is inhibited by pegylated arginine deiminase (ADI-PEG20)

Natalie Burrows, Gaelle Cane, Mathew Robson, Edoardo Gaude, William Howat, Peter W Szlosarek, R Barbara Pedley, Christian Frezza, Margaret Ashcroft and Patrick H Maxwell

Supplementary tables, figures and references

Supplementary table 1: List of primary antibodies used

Protein target (human unless specified)	Manufacturer/ Catalogue number	Species raised in	Application	Dilution
ASL	Santa Crus Biotechnology/ sc-1166787	Mouse monoclonal	Western-blotting	1: 250
ASS1	Abcam/ ab170952	Rabbit monoclonal	Western-blotting	1:500
β-actin	Sigma-Aldrich/ A2228	Mouse monoclonal	Western-blotting	1: 80 000
BrdU	Abcam/ ab1893	Sheep polyclonal	IHC	1: 500
CA-IX	M75/ gift from J.Pastorek , Bratislava (1)	Mouse monoclonal	Western-blotting	1: 10
CD31 (mouse)	BD Pharmingen/ 553370	Rat monoclonal	IF tissue	1:50
Cleaved caspase-3	Cell Signaling Technologies/ 9664	Rabbit monoclonal	IHC	1:100
eIF-2α	Cell Signaling Technologies/ 2103	Mouse monoclonal	Western-blotting	1:1000
eIF-2α-pS51	Abcam/ ab32157	Rabbit monoclonal	Western-blotting	1:1000
HIF-1α	Novus/ NB100-479	Rabbit polyclonal	Western-blotting	1:500
HIF-2α	Novus/ NB100-122	Rabbit polyclonal	Western-blotting	1:500
iNOS	Santa Cruz Biotechnology/ sc-7271	Mouse monoclonal	Western-blotting	1:250
Isotype control: Rabbit IgG	Vector Laboratories/ I-1000	Rabbit	IHC	Assay dependent
Isotype control: Mouse IgG1	Abcam/ ab18443	Mouse	IHC	Assay dependent
mTOR	Cell Signaling Technologies/ 2972	Rabbit polyclonal	Western-blotting	1:1000
mTOR-pS2481	Cell Signaling Technologies/ 2974	Rabbit polyclonal	Western-blotting	1:1000
Nitrotyrosine	Millipore/ 06-284	Rabbit polyclonal	IHC	1:100
Pimonidazole	Natural Pharma/ Hydroxyprobe clone 4.3.11.3	Mouse monoclonal	IF tissue/ IHC	1: 50
p70 S6 Kinase	Cell Signaling Technologies/ 9202	Rabbit polyclonal	Western-blotting	1:1000
p70 S6 Kinase-pT389	Cell Signaling Technologies/ 9205	Rabbit polyclonal	Western-blotting	1:1000

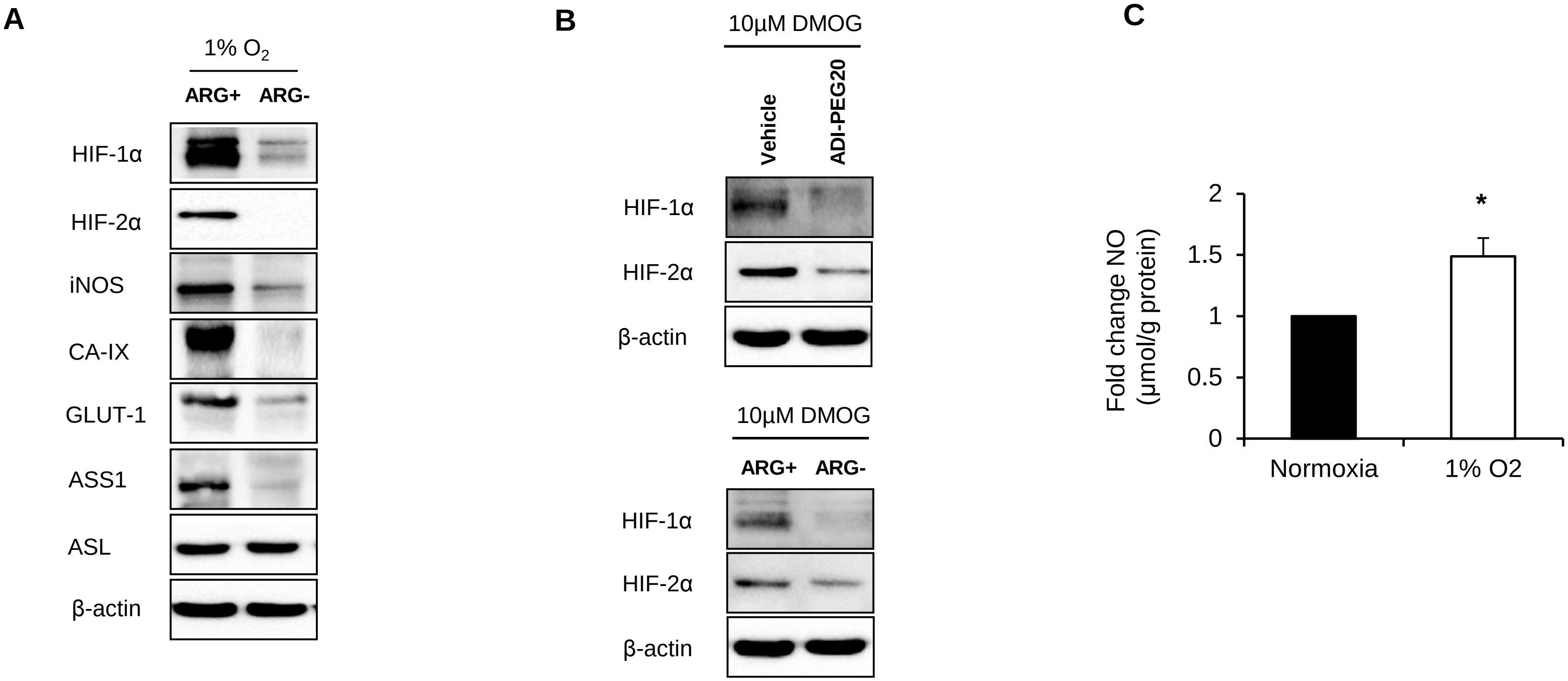

Supplementary figure 1. A: HCT116 cells cultured for 24h under 1% O_2 in arginine free (ARG-) SILAC media, show reduced expression of HIF-1α, HIF-2α and downstream targets iNOS, CA-IX and GLUT-1 compared to cells cultured in SILAC media supplemented with arginine (ARG+). ASS1 was also decreased in ARG- media. Blot is representative of 5 experiments. **B:** HIF-α protein expression is reduced in the presence of 5mU/ml ADI-PEG20 or in arginine free media (ARG-), in HCT116 cells treated for 24h with 10μM dimethyloxaloylglycine (DMOG). Blot is representative of 2 experiments. **C:** NO increases under hypoxia (*$p<0.05$). The concentration of extracellular NO was determined from the enzymatic reduction of nitrate, a stable metabolite of NO in media from cells incubated in normoxia/hypoxia for 24h. Data represents the mean ± S.E.M. of 4 experimental repeats. Extracellular NO concentrations were determined using the Total Nitric Oxide and Nitrate/Nitrite Assay kit (R&D Systems Europe Ltd, Abingdon, UK).

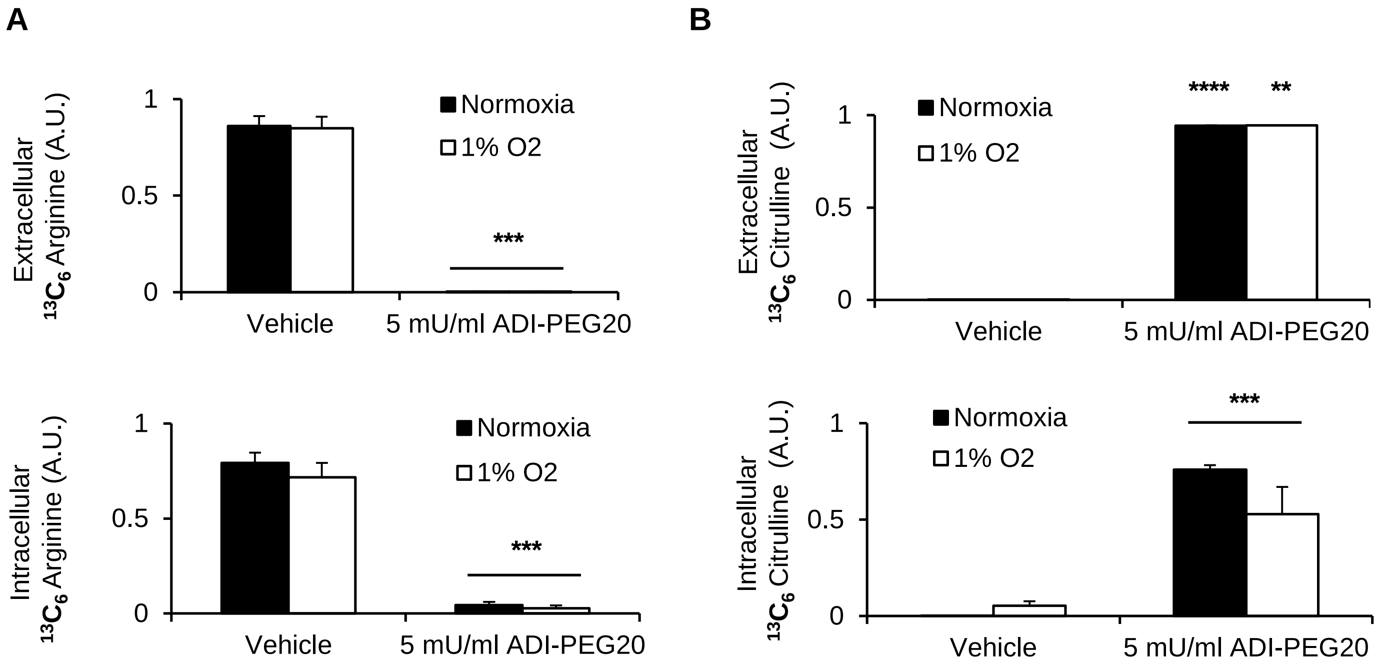

Supplementary figure 2. 5mU/ml ADI-PEG20 degrades extracellular arginine and depletes intracellular arginine A: Stable isotopologue tracing revealed that 5mU/ml ADI-PEG20 depleted extracellular $^{13}C_6$ arginine by 99.7% under normoxia/hypoxia (***p<0.0001). Intracellular $^{13}C_6$ arginine was similarly reduced by 94.4 ± 5.6% S.E.M. (normoxia) and 96.2 ± 5.2% S.E.M. (hypoxia: ***p<0.0001). **B:** ADI-PEG20 converts arginine into citrulline and ammonia: In ADI-PEG20 treated cells, extracellular $^{13}C_6$ citrulline is increased under normoxia and hypoxia (**p<0.001, ****p<0.00001). Intracellular $^{13}C_6$ citrulline was similarly increased (***p<0.0001). This is likely due to internalization of $^{13}C_6$ citrulline, occurring in the presence of a high extracellular concentration. It is important to note that increased intracellular citrulline does not inhibit iNOS activity (2). Cells were incubated with $^{13}C_6$ arginine for 24h and the isotopologue distribution of $^{13}C_6$ arginine and $^{13}C_6$ citrulline assessed. Data represents the mean ± S.E.M. of 3-4 experiments.

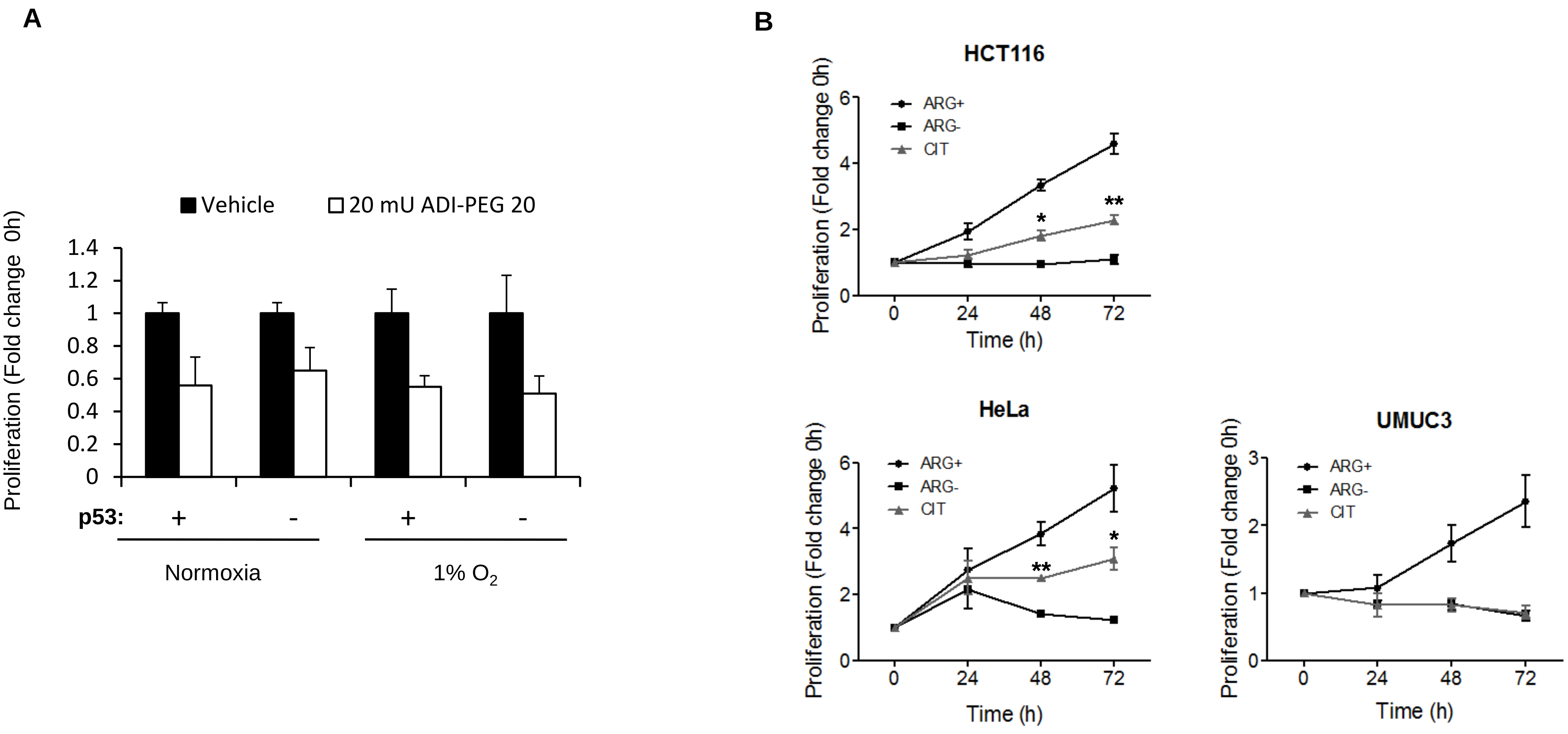

Supplementary figure 3A: Effects of ADI-PEG20 on proliferation do not differ between parental and p53-/- cells. Parental and p53-/- HCT116 cells (3) were cultured in media supplemented with vehicle (PBS) or 20 mU/ml ADI-PEG20 under normoxia or 1% O_2 for 48h. **B: In arginine-free media, HCT116 cell proliferation is rescued in the presence of citrulline and ammonium chloride (NH_4Cl).** In CIT media, cell proliferation was significantly increased in HCT116 and HeLa cells after 48 and 72h (*$p<0.01$, **$p<0.001$ versus ARG- media). No effect was seen on UMUC3 cells cultured in CIT media. Cells were cultured for up to 72h in the following SILAC media: ARG+ (media supplemented with arginine), ARG- (arginine-free media) or CIT (media supplemented with 1mM citrulline and 1mM NH_4Cl: Concentrations used are stoichiometrically equivalent to the amount of arginine in ARG+ medium). Data represents the mean ± S.E.M. of 3-4 independent experiments. For A-B, proliferation was assessed via MTT assay (see methods, main text). Fold induction of proliferation relative to 0h is shown.

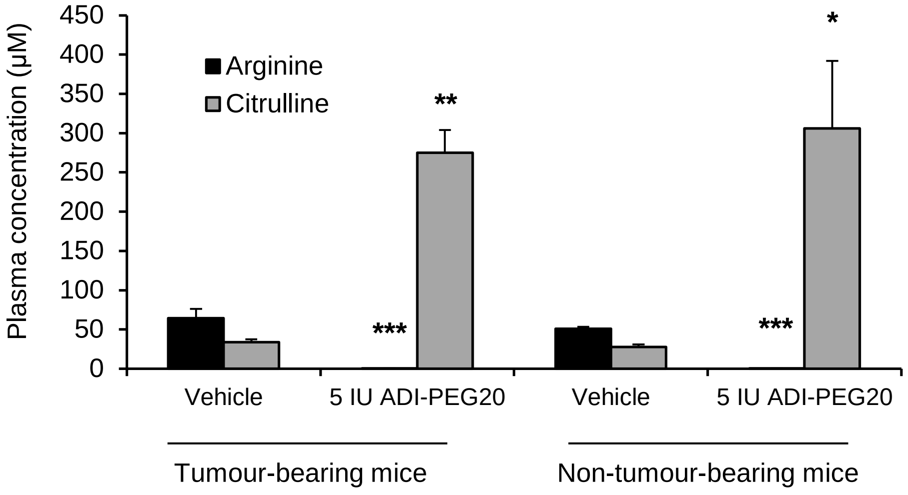

Supplementary figure 4. Metabolomic analysis of arginine and citrulline plasma levels from tumour-bearing and non-tumour bearing mice. Mice treated with ADI-PEG20 had significantly lower plasma arginine levels (***p<0.000001) and significantly higher citrulline levels (*p<0.05, **p<0.001), due to ADI-PEG20 mediated conversion of arginine to citrulline. Size matched tumours were treated with vehicle (PBS) or 5IU ADI-PEG20. Methods used are described in (4). Briefly, 100µl mice plasma samples were extracted with 300µl ice-cold methanol containing the internal standards (13C6 L-arginine and D4 L-citrulline). After centrifugation, methanolic extracts were evaporated to dryness. Then, dried extracts were reconstituted in 85% acidified acetonitrile and injected into the UPLC-MS/MS system. Analytes were resolved using an Accela UPLC (Thermo Scientific, UK) equipped with 1.7 µm HILIC Kinetex 2.1 × 50mm UPLC column (Phenomenex, UK) and a mobile phase gradient of buffer A (water + 0.1% formic acid) and buffer B (acetonitrile + 0.1 formic acid) at a flow rate of 250 µl/min. Eluting compounds of interest were detected using a TSQ Vantage mass spectrometry system (Thermo Scientific, UK). The optimum transitional daughter ions mass of each analyst were as follows: arginine m/z 175.1 → 70.2, citrulline m/z 176.1 → 70.2, 13C6 arginine m/z 181.0 → 74.2 and D4 citrulline m/z 180.2 → 74.2. Data represents the mean ± S.D. of 5 tumour-bearing and 3 non-tumour bearing mice per treatment group.

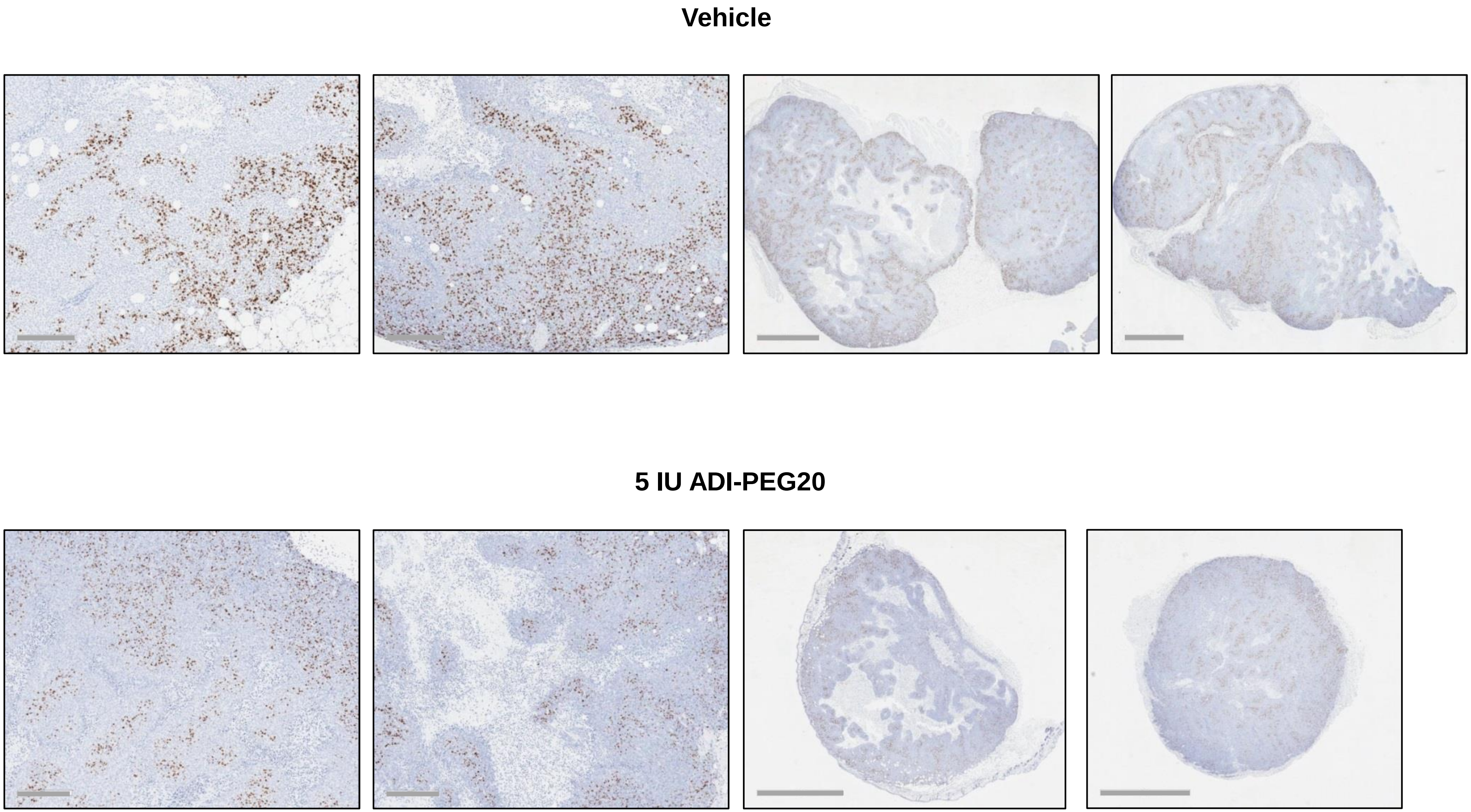

Supplementary figure 5A. Representative images of BrdU staining in vehicle and 5IU ADI-PEG20 treated tumours from data described in Figure 4. Scale-bar represents 300µm (4 images on left) and 2mm (4 images on right).

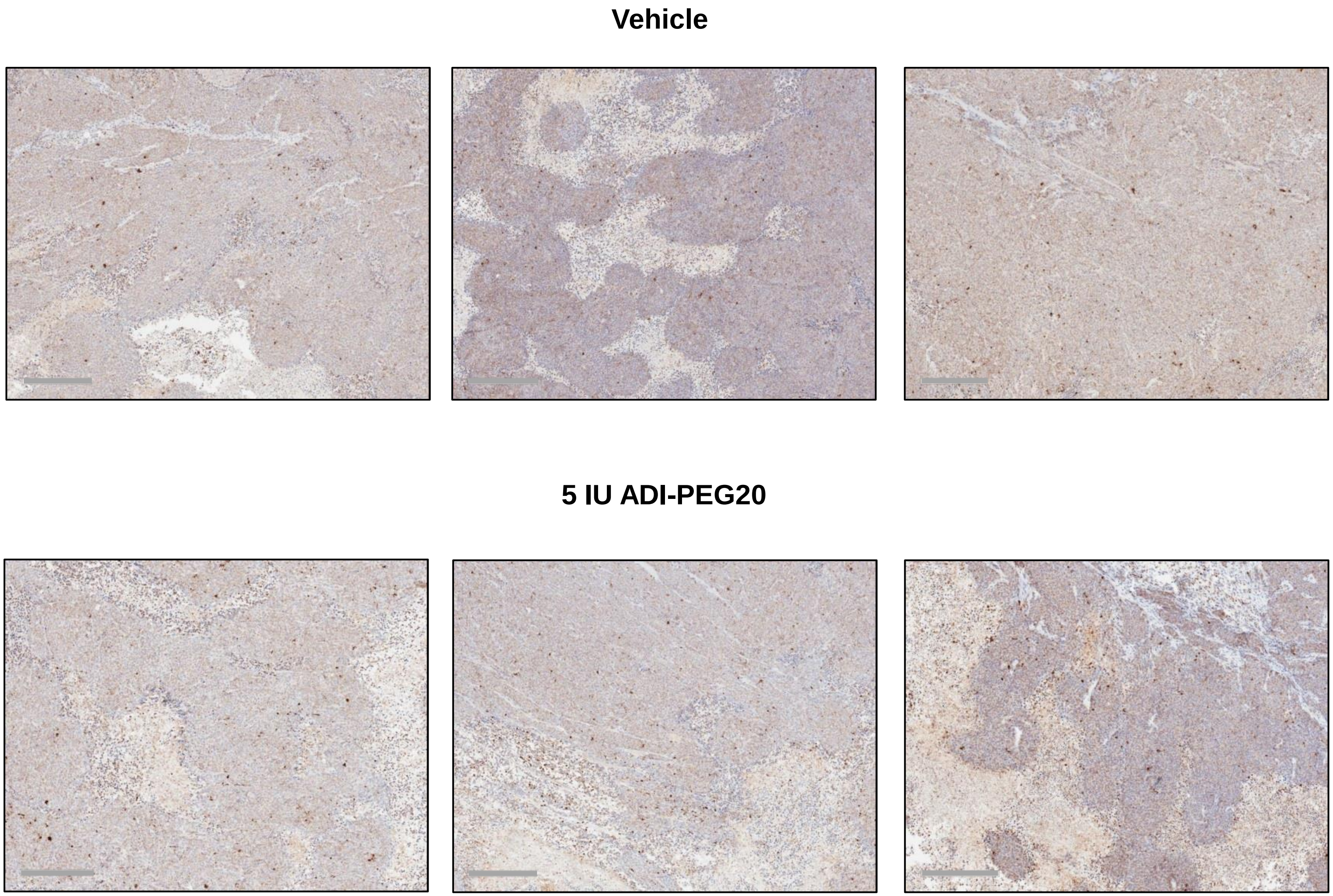

Supplementary figure 5B. Representative images of cleaved casapse-3 staining in vehicle and 5IU ADI-PEG20 treated tumours from data described in Figure 4. Scale-bar, 300μm.

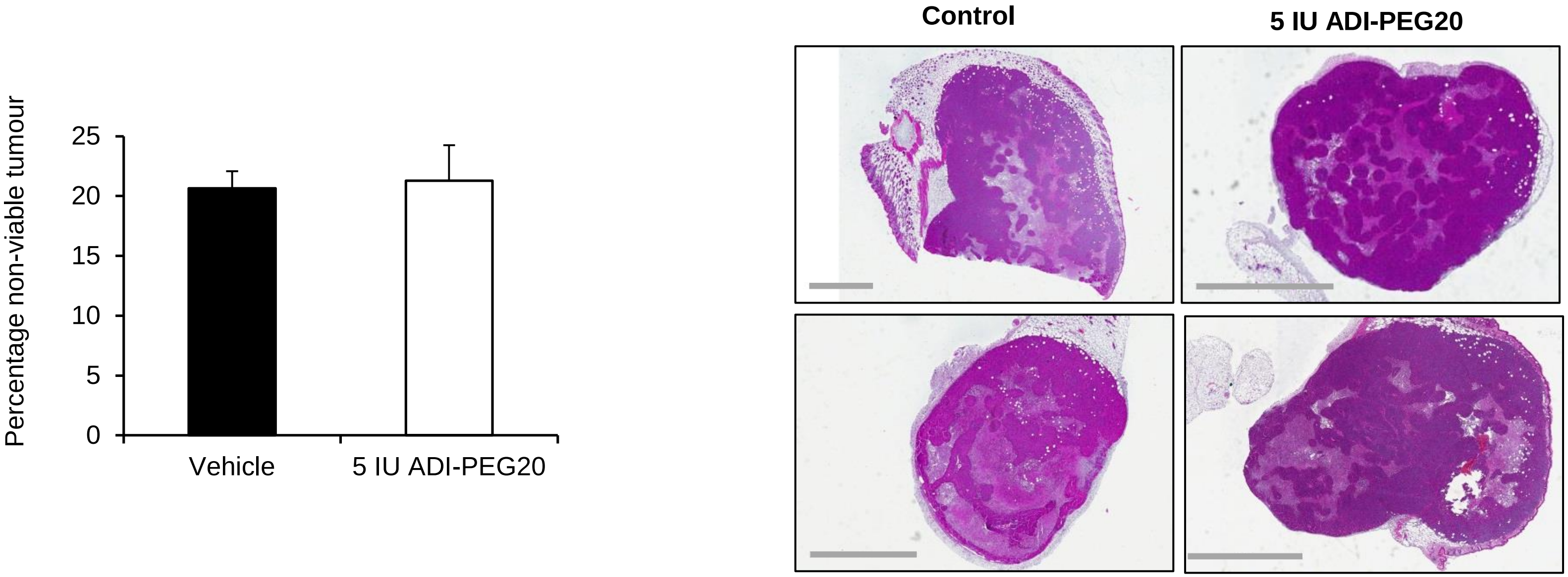

Supplementary figure 6. Necrotic fraction did not differ between control and ADI-PEG20 treated tumours. Graphical representation of percentage non-viable tumour and representative haematoxylin and eosin (H and E) stained images are shown. Percentage non-viable tumour was calculated from H and E stained tumour sections using Aperio/Spectrum v10.2.2.2317 software, where total section area and total necrotic area was determined. Data represents the mean ± S.E.M. of 5 mice per treatment group. Scale-bar, 2mm.

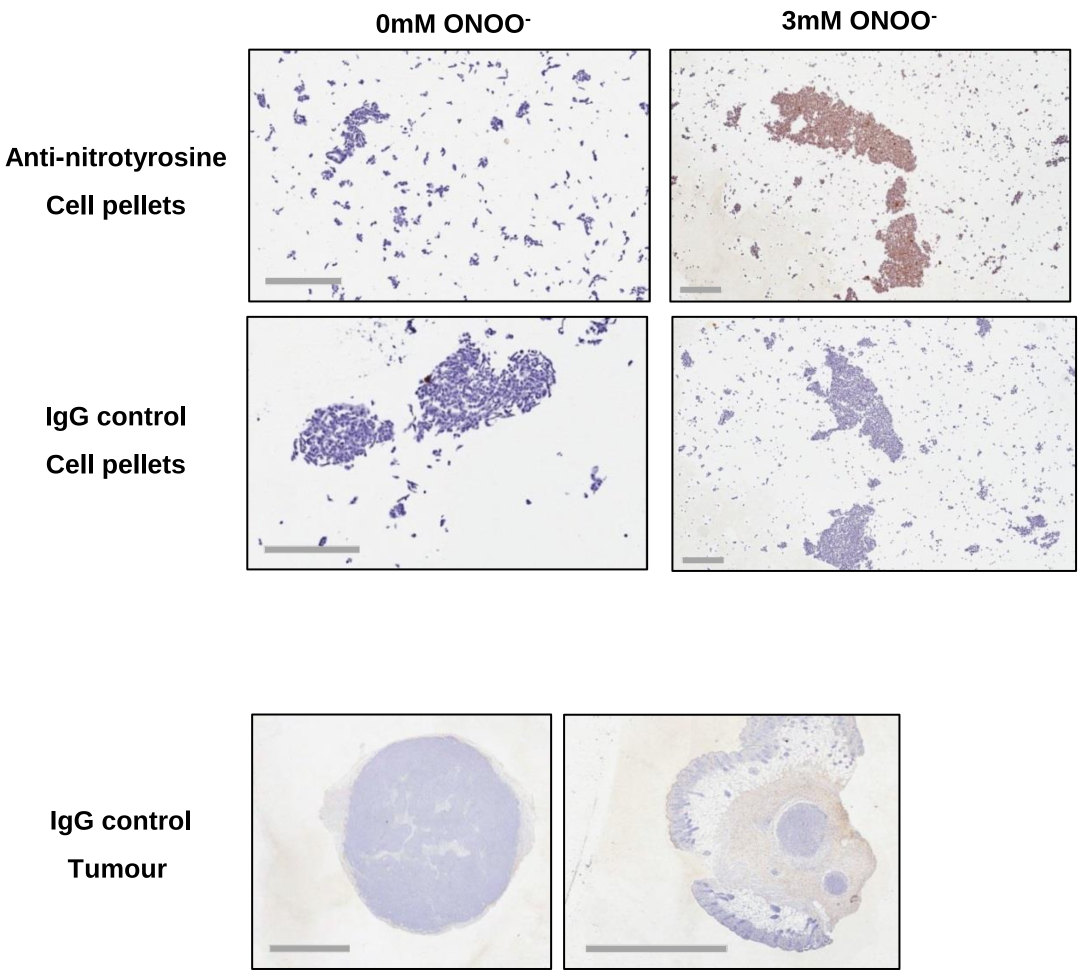

Supplementary figure 7: Cell pellets were prepared from HCT116 cells treated with 3 mM peroxynitrite ($ONOO^-$) for 10min. Peroxynitrite nitrates protein tyrosine residues and was thus used as a positive control for nitrotyrosine antibody specificity. Top panel; cell pellet sections stained for nitrotyrosine, treated with or without 3mM $ONOO^-$. Only treated cell pellets stained positive for nitrotyrosine (brown). Middle and bottom panel; Cell pellet sections and HCT116 tumour sections stained with a rabbit IgG concentration-matched, isotype control. Scale-bar top panel: 400µm, bottom panel, 2mm.

References

1. Zat'ovicova, M. et al. Monoclonal antibodies generated in carbonic anhydrase IX-deficient mice recognize different domains of tumour-associated hypoxia-induced carbonic anhydrase IX. Journal of immunological methods 282, 117-134 (2003).
2. Rogers, N. E. & Ignarro, L. J. Constitutive nitric oxide synthase from cerebellum is reversibly inhibited by nitric oxide formed from L-arginine. Biochemical and biophysical research communications 189, 242-249 (1992).
3. Bunz, F. et al. Requirement for p53 and p21 to sustain G(2) arrest after DNA damage. Science 282, 1497-1501, doi:DOI 10.1126/science.282.5393.1497 (1998).
4. Allen, M. D. et al. Prognostic and therapeutic impact of argininosuccinate synthetase 1 control in bladder cancer as monitored longitudinally by PET imaging. Cancer research 74, 896-907, doi:10.1158/0008-5472.CAN-13-1702 (2014).
